# Current News

**Published:** 1890-07

**Authors:** 


### Current News.

### AMERICAN DENTAL ASSOCIATION.

THE Thirtieth Annual Session of the American Dental Association will be held at Excelsior Springs, Missouri, commencing Tuesday, August 5, 1890, at 10 o'clock, A.M.

GEO. H. CUSHING,
*Recording Secretary.*

### MEETING OF THE AMERICAN DENTAL ASSOCIATION.

THE railroad arrangements are not all completed, but enough is known to assure, at least, the usual reduction of one and a third fare, on certificate plan, by all the different passenger associations.

Arrangements are being made for a special train from Chicago, which will leave here Sunday afternoon and reach Excelsior Springs Monday morning. This will give the entire day, Monday, for the different sections to complete their reports. All parties wishing to go on this special train will confer a favor by letting us know at once, so that we may know how many to arrange for. Just what rate will be secured for the round trip is not definitely settled, but we expect a low one. A notice will be issued later giving exact time of starting and route selected. Application has been made for reduced rates for the four associations,—The American Dental Association, College Faculty Association, National Board of Dental Examiners, and The Dental Protective Association. We are trying to get this rate good for ten days, so that we need not adjourn until everything is finished.

Parties purchasing tickets should be sure to get receipt, showing that they have paid full fare going, which will enable them to get return tickets for one-third regular fare.

J. W. CROUSE,
*Chairman Excursion Committee.*

2231 PRAIRIE AVENUE, CHICAGO.

THE "germ-theory" is thus defined by Dr. Wardlaw: Particles of food, mucus, or saliva, wedged between the teeth or hid away in some fissure, or being in some depression, or adhering to some protected surface of a tooth, and not removed by friction, nor dissolved nor washed away by the fluids of the mouth, are taken possession of by bacteria, and fermentation begins; lactic acid, the effete matter of the microbes, is formed, and immediately unites with the lime of the enamel; a slight depression is here made, giving more secure lodgement for additional food, and further fermentation continues. The enamel armor is thus gradually pierced, and the less dense dentine is exposed: ingress is had by the bacteria; they consume the organic elements of the dentine; lactic acid is secreted more abundantly; it presses forward in advance, breaking down the walls of the tubuli; the mirococci follow closely on, crowding in and packing full the tubuli; a secure foothold having been obtained, destruction runs riot; softening and breaking down proceeds in all directions, and we have a typical case of "caries."

---

DR. S. A. WHITE treats fistulous opening from the outside, in ninety-nine cases out of a hundred one thorough injection of pure creosote and iodine being found sufficient; the root is filled at the same sitting, and usually in a week no trace of the fistula can be found.

---

PENNSYLVANIA STATE DENTAL SOCIETY.—The twenty-second annual meeting of the Pennsylvania State Dental Society will meet at Gettysburg, Pa., on Tuesday, July 29, 1890, at 10 A.M., session to continue three days.

C. V. KRATZER,
*Recording Secretary.*

---

THE NATIONAL ASSOCIATION OF DENTAL FACULTIES.—The National Association of Dental Faculties will meet on Monday, August 4, 1890, at 10 A.M., at Excelsior Springs, Missouri.

JOHN S. MARSHALL, *Secretary.*

---

THE dentist can do more and better work by sitting at the operating-chair. I use an ordinary office screw-chair.

W. C. BROWNE.

